# Veratrum Viride in Pericarditis

**Published:** 1869-04

**Authors:** J. Waring-Curran

**Affiliations:** The Practitioner


Veratrum Viride in Pericarditis.—By J. Waring-Curran, L. K. Q. C. P. I., in *The Practitioner*, August.

Dr. J. Waring-Curran states that he has found this drug of the highest value in the treatment of pericarditis. The extract made by inspissating the juice of the root is the preparation he has invariably employed, prescribing it in two-grain doses, with one grain of calomel in the form of pill, every two hours, and carefully watching its effects. Its power of reducing the frequency of the pulse, and of increasing the renal and hepatic secretions, lead him to regard the veratrum viride as almost a specific for pericarditis. In cases of acute rheumatism in which pericardial symptoms began to be manifest, he feels assured "that the mischief was baffled by the early and careful exhibition of ten-drop doses of the tincture of veratrum viride in the asthmatic mixture."

